# Improvement of Thermal Stability via Outer-Loop Ion Pair Interaction of Mutated T1 Lipase from *Geobacillus zalihae* Strain T1

**DOI:** 10.3390/ijms13010943

**Published:** 2012-01-17

**Authors:** Rudzanna Ruslan, Raja Noor Zaliha Raja Abd. Rahman, Thean Chor Leow, Mohd Shukuri Mohamad Ali, Mahiran Basri, Abu Bakar Salleh

**Affiliations:** 1 Enzyme and Microbial Technology Laboratory, Faculty of Biotechnology and Biomolecular Sciences, Universiti Putra Malaysia, 43400 UPM Serdang, Selangor, Malaysia; E-Mails: rudzanna@gmail.com (R.R.); adamleowupm1@gmail.com (T.C.L.); shukuri12@gmail.com (M.S.M.A.); abubakar67@yahoo.com (A.B.S.); 2 Institute of Bioscience, Universiti Putra Malaysia, 43400 UPM Serdang, Selangor, Malaysia; E-Mail: mahiran@science.upm.edu.my; 3 Faculty of Science, Universiti Putra Malaysia, 43400 UPM Serdang, Selangor, Malaysia

**Keywords:** thermostable lipase, ion pair interaction, thermal stability, CD spectral analysis, lipase crystal, X-ray diffraction

## Abstract

Mutant D311E and K344R were constructed using site-directed mutagenesis to introduce an additional ion pair at the inter-loop and the intra-loop, respectively, to determine the effect of ion pairs on the stability of T1 lipase isolated from *Geobacillus zalihae*. A series of purification steps was applied, and the pure lipases of T1, D311E and K344R were obtained. The wild-type and mutant lipases were analyzed using circular dichroism. The *T_m_* for T1 lipase, D311E lipase and K344R lipase were approximately 68.52 °C, 70.59 °C and 68.54 °C, respectively. Mutation at D311 increases the stability of T1 lipase and exhibited higher *T_m_* as compared to the wild-type and K344R. Based on the above, D311E lipase was chosen for further study. D311E lipase was successfully crystallized using the sitting drop vapor diffusion method. The crystal was diffracted at 2.1 Å using an in-house X-ray beam and belonged to the monoclinic space group *C2* with the unit cell parameters *a* = 117.32 Å, *b* = 81.16 Å and *c* = 100.14 Å. Structural analysis showed the existence of an additional ion pair around E311 in the structure of D311E. The additional ion pair in D311E may regulate the stability of this mutant lipase at high temperatures as predicted *in silico* and spectroscopically.

## 1. Introduction

Many lipases preserve their activity at extreme conditions, such as temperature and pH. Lipases have many industrial applications, for example in the processing of agroindustrial residues [1], leather manufacturing, detergents, and flavor production in dairy and medical applications [2]. Mostly, *Bacillus* lipases display diverse selectivity to the chain length of the acid, and few enzymes show positional specificity but several enzymes can be applied in pharmaceutical industry due to the enantioselectivity [3].

To meet the industrial demand, a high activity and heat-stable lipase is preferred to mediate catalysis at high temperature. Thermal stability is a major requirement for a commercial enzyme because thermal denaturation is a common cause of enzyme inactivation [4]. In the previous decade, protocols based on *in vitro* screening of large populations of protein variants, which are collectively known as directed evolution methods, have led to extraordinary success in altering the enzymatic properties, such as stability, affinity and selectivity of proteins [5]. Nevertheless, to improve industrial biocatalyst features, methods of chemical modification and immobilization of enzymes have been considered and chemical modifications made, such as stabilizing additives [6,7]. Furthermore, enzyme engineering via immobilization techniques is perfectly compatible with other chemical or biological approaches to improve enzyme functions [8].

Rational design has been applied in many fields of mutagenesis study. The extensive and systematic testing of each product of the executable code is needed to describe and support the importance of the research. An important point of focus is to determine which mutation will affect the stability of the mutant with respect to the wild-type. The mutants are mainly based on the development of different energy functions and are suited to compute the stability free energy changes [9,10]. In this proteomic era, the mutagenic process is constructed using many developed methods. The development of predictors is needed to study the effects of the mutation computationally before conducting experiments. These have been used in many fields of research. The molecular modeling and site-directed mutagenesis can be used to elucidate the structural basis [11]. Moreover site-directed mutagenesis, directed evolution, allows fine modification of the properties of their lipases such as introducing a new ion pair into the structure [3]. Rahman *et al* [12]. have shown that ion pair networks play a key role in maintaining enzymatic stability at extreme temperatures.

Most lipases contain a lid domain controlling access to the active site [13]. Lipase activity is greatly increased at the lipid-water interface which is known as interfacial activation [14]. The interaction of the enzyme with the lipid aggregates induces the displacement of the lid, which makes the active site accessible to individual substrate molecules and increases the catalytic activity [15]. Unlike T1 lipase, the crystal structure of BTL2 was solved in an open conformation with two molecules of triton detergent present in the active site.

Here, we report on the effect of an ion pair network on the stability of T1 lipase by introducing an additional ion pair at the inter-loop and the intra-loop. In addition, we elucidated the structure of D311E lipase to identify the additional interactions that govern the stability of this mutant.

## 2. Results and Discussion

### 2.1. Rational Design of Mutant Lipases

The crystal structure of thermostable T1 lipase (PDB ID: 2DSN) contained the metal ions Ca^2+^, Zn^2+^, Cl^−^ and Na^+^ as well as chain A and chain B in asymmetry [16]. The structure solved in closed conformation with the active site buried under a long lid-helix. Based on this crystal structure, the mutant D311E lipase and K344R lipase were designed to locate the inter-loop and intra-loop interactions, respectively, by introducing additional non-bonded interactions. These positions were chosen to compare and study the effect of the ion pair and the hydrogen bond that was formed after mutation.

**Figure 1:**
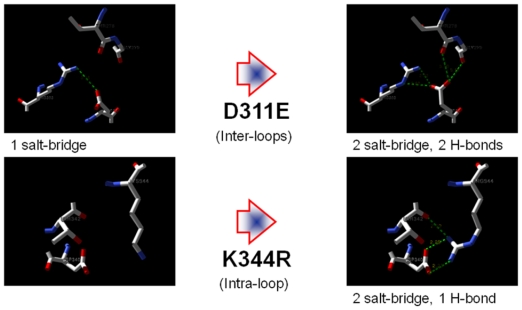
Protein tailoring of D311E lipase forming inter-loops networking, whereby K344R forming intra-loop networking. The green dash line indicated ion pair and hydrogen bond interactions.

Figure 1 shows the ion pair and hydrogen bond interactions that may be introduced into the D311E lipase and K344R lipase structure and were analyzed using Swiss PDB Viewer. The images were generated using POV-Ray in order to obtain high quality images. This interaction indicated that ion pair interactions were located at exposed parts of the inter-loops and the intra-loops of the protein. More ion pairs were observed in D311E lipase compared to those in K344R lipase. These interactions may provide stability to the D311E lipase structure due to the high flexibility of the outer portion of the protein compared to that of the inner portion of the protein. In addition, this method was used in previous research that described the success of the rational design approach in improving the stability of the mutant [17–19].

### 2.2. Prediction of Protein Stability Changes upon Point Mutation

I-Mutant 2.0 predictions were performed using the protein structure or, more importantly, from the protein sequence. In general, an exposed residue with an increased stability is free to mutate compared to a partially buried residue.

Table 1, summarizes the prediction result of the protein stability that changes upon point mutation. The D311E protein sequence was submitted to I-Mutant 2.0 software as an input file. This software predict minima at the verified recombination sites-supporting the assumption to cut at less sensitive regions (high acceptance of substitutions) [20]. The stability of D311E lipase was expected to increase because we obtained a positive free energy change (DDG) value, which increased the stability. The substitution of D311 to E311 improved the structural properties by introducing the interactions of the additional ion pair. The additional ion pair strengthened the structural interaction of the D311E lipase.

The RI value (Reliability Index) is computed when the sign of the stability change is predicted and evaluated based on the output of the SVM (support vector machine) at *O* as RI = 20 times to absorbance (0–0.5) [21]. The RI of this protein was 7.0 kcal/mole. A high RI is important to interpret the output data, as it indicates the probability that the structure will not fail to perform stabilizing functions [1]. The predicted optimal Relative Solvent Accessible Area (RSA) value of D311E lipase was 67.3%. The RSA value is calculated using the DSSP program when the prediction is based on the enzymatic structure, by dividing the accessible surface area value of the mutated residue by the free residue surface [22]. DSSP program has also been used by [23] Capriotti *et al*. to calculate the RSA of the 21^st^ element vector of the protein structural environment. With those values, we expect that this prediction is a good indicator and useful in studies on the inter-relationships of D311E lipase structure and energetic measurements.

### 2.3. Circular Dichroism Analysis

The CD spectra of D311E lipase were analyzed as a function of temperature at 220 nm. The wavelength 220 nm was set to monitor the transition of α-helices to disordered structures because they exhibited characteristic signals at this wavelength. The purified lipases of T1, D311E and K344R were expressed using the pGEX/T1S vector and purified at 4.6-fold, 1.98-fold and 4.0-fold, respectively.

**Figure 2:**
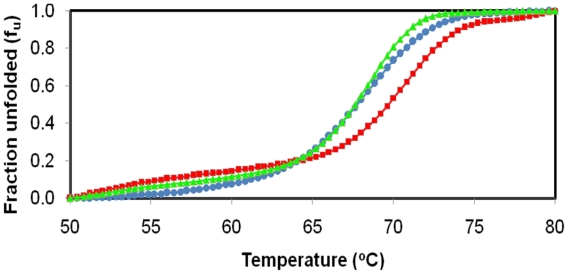
Denatured protein analysis by circular dichroism. *T_m_* of T1 lipase (blue) was at ~68.52 °C, K344R lipase (green) at ~68.54 °C and D311E lipase (red) at ~70.59 °C.

Figure 2 shows that the analysis of the unfolded protein of D311E lipase compared with another mutant, K344R lipase and its native enzyme, T1 lipase. The melting temperatures (*T_m_*) of the three proteins were different with variant unfolded fractions. All of the proteins started to unfold at 60 °C at different melting temperature. These differences might be due to loss of the protein secondary structure followed by an increase in the unordered conformations of the proteins. T1 lipase (circle), K344R lipase (triangle) and D311E lipase (square) showed different *T_m_*, which were 68.52 °C, 68.54 °C and 70.59 °C, respectively. D311E lipase was the most stable among the three proteins. D311E lipase involved a different portion of the loop to significantly strengthen the interaction compared to mutant K344R, in which the interaction was found solely inside the loop region.

Changes in protein stability were determined upon making point mutations with I-mutant 2.0. The mutants D311E (exposed residue) and K344R (moderately exposed residue) showed increased and decreased stability, respectively. Despite a reduction of one ion pair, networks formed by the ion pair and the hydrogen bond between loops were stronger than the interactions located inside the loop of the crystal structure of T1 lipase. As suggested by [13] Derewenda *et al*., the converted structure is stabilized once proper inter-subunit bridges are formed. Based on Figure 2, the K344R mutant had the same *T_m_* as the wild-type. The ion pair introduced did not affect the thermal stability.

**Figure 3:**
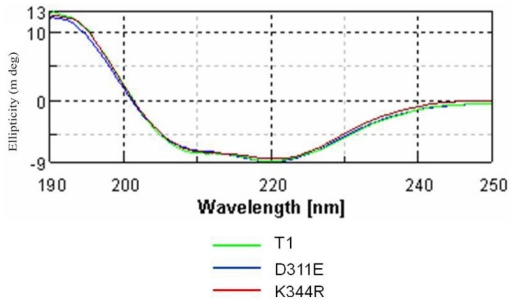
CD spectra of T1 lipase (green) and mutant D311E (blue) and K344R (red).

The CD spectra (molecular ellipticity) of T1 lipase and its mutants were analyzed as a function of temperature at 220 nm to monitor the transition of α-helical structures to disordered structures. As shown in Figure 3, there are no significant structural changes observed for T1 lipase and its mutants. The result indicated that the mutation at loop regions did not change the conformation of the proteins. However, there is an increase of *T_m_* for mutant D311E as compared to wild-type T1 lipase and another mutant K344R. When a protein starts to unfold due to heating, the process will go through an intermediate state, thus the free energy change *(ΔG*) at equilibrium was zero. As a consequence, the T1 lipase, D311E and K344R showed a melting temperature, *T_m_* of 68.52 °C (341.52 K), 70.59 °C (343.59 K) and 68.54 °C (341.54 K), respectively with the unfolding enthalpy (*ΔH*) and entropy (*ΔS*) as listed in Table 2.

Although the prediction of K344R mutant stability that was generated by the I-Mutant 2.0 software showed a decrease in stability (Table 1), the experimental CD results showed no change in stability. Because the prediction and CD data indicated that D311E was a better enzyme than K344R; D311E was chosen for structural analysis.

### 2.4. Effect of Temperature on D311E and T1 Lipase Activity and Stability

**Figure 4:**
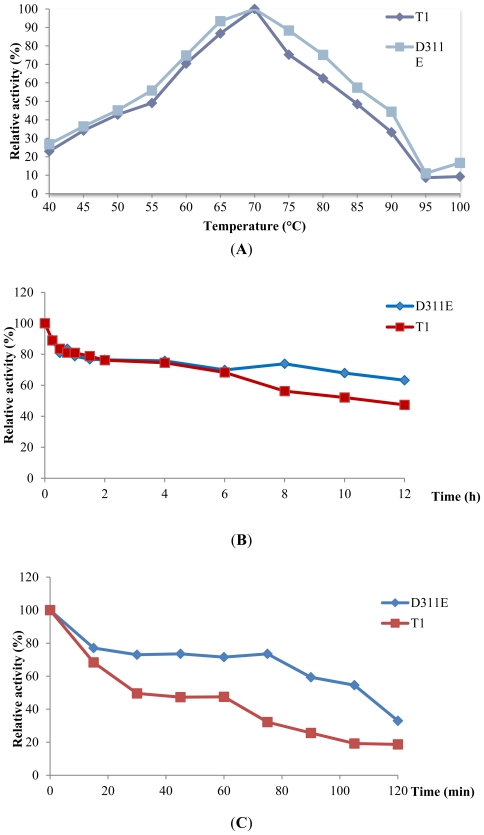
Effect of temperature on D311E and T1 lipase activity and stability. (**A**) The optimum temperature of D311E and T1 lipase; (**B**) Effect of temperature on lipase stability, pre-incubated at 70 °C; (**C**) Effect of temperature on lipase stability, pre-incubated at 60 °C.

Both D311E and T1 had an optimum temperature of 70 °C (Figure 4A) for stability as shown in Figures 4B,C, the *t_1/2_* of T1 lipase at 60 °C and 70 °C was 30 min and 10 h, respectively. However, the mutant D311E lipase enhanced the temperature effect as compared to the wild-type. The *t_1/2_* of D311E lipase at 60 °C and 70 °C was 110 min and up to 12 h, respectively. A single mutation of LST-03 lipase from *Pseudomonas aeruginosa* LST-03 was found to stabilize the lipase by inducing structural changes including the formation of a salt bridge and hydrogen bonds [24]. Moreover, [25] Vetriani *et al*. suggest that ion-pair networks may provide a general strategy for manipulating enzyme thermostability of multisubunit enzymes. This shows that the introduction of ion pair stabilized the T1 lipase at high temperature. Most importantly, the ability to retain activity and stability at high temperatures demonstrates great potential in industrial fields.

### 2.5. Crystallization of D311E lipase

**Figure 5:**
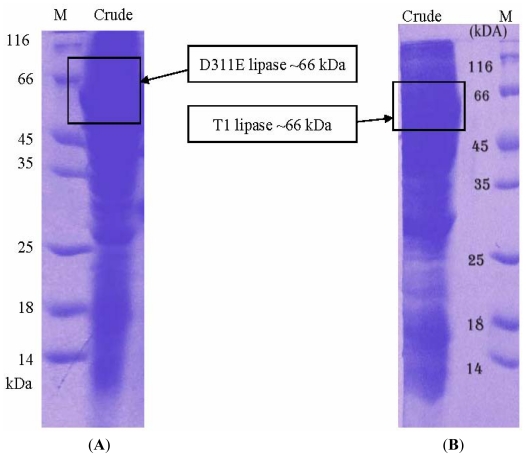
(**A**) SDS-PAGE (12%) of crude mutant lipase D311E. M: standard protein markers (kDa); (**B**) SDS-PAGE (12%) of crude native T1 lipase. M: standard protein markers.

The purified D311E lipase was assayed using the colorimetric assay [26], and the purity was checked using SDS-PAGE and native PAGE [27]. D311E lipase was successfully purified to 10.56-fold with 15.71% of yield using a serial-step chromatographic strategy (Table 3). The amount of the total protein (mg) and total activity (U) of D311E lipase were two times higher than the wild-type (Data not shown). Figure 5 shows the crude sample D311E lipase (A) loaded on SDS-PAGE as compared to the crude of wild-type T1 lipase (B). The estimated size (66 kDa) was similar to its predicted molecular weight but the band of D311E lipase was slightly thicker than T1 lipase obtained through SDS-PAGE.

**Figure 6:**
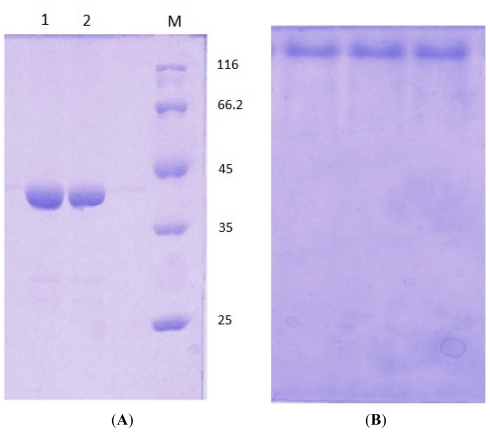
(**A**) SDS-PAGE (12%) of mutant lipase D311E. M: standard protein markers (kDa); purified lipase (lane 1 and 2); (**B**) Single band of D311E lipase on Native PAGE analysis after ion exchange chromatography.

For crystallization, ion exchange chromatography was performed as the final step of purification for D311E lipase. A single band was observed in SDS-PAGE (Figure 6A), estimated to be 43 kDa and native–PAGE (Figure 6B), indicating that this purified D311E lipase was suitable for protein crystallization.

**Figure 7:**
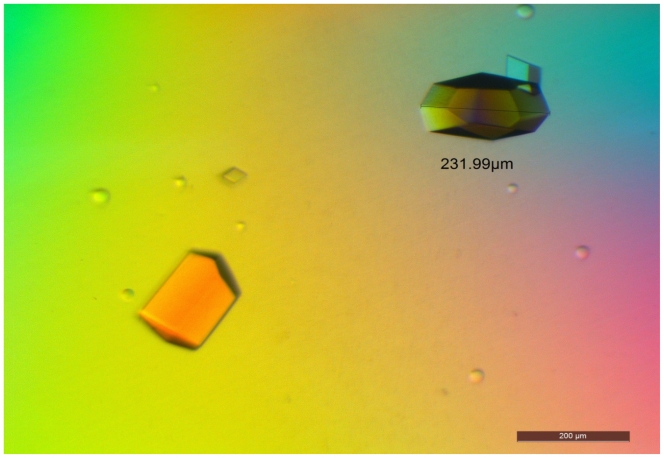
D311E lipase crystal grown using sitting drop method with 0.1 M MES pH 5.5, 0.1 M Sodium phosphate, 0.1 M Potassium phosphate, and 1.5 M NaCl as precipitant reagent.

Optimization of D311E lipase crystallization was conducted and it was found that the best formulation was 0.1 M MES pH 5.5, 0.1 M sodium phosphate, 0.1 M potassium phosphate, and 1.5 M NaCl as a precipitant. Crystallization conditions such as pH, protein concentration, precipitant, and temperature were found to affect the quantity, size and quality of the crystal. The crystals were grown using the sitting drop vapor diffusion method. Good quality crystals of D311E lipase were observed after overnight incubation at 20 °C. The size of the crystal reached 0.2 mm × 0.1 mm × 0.1 mm (Figure 7).

### 2.6. X-Ray Data Collection

**Figure 8:**
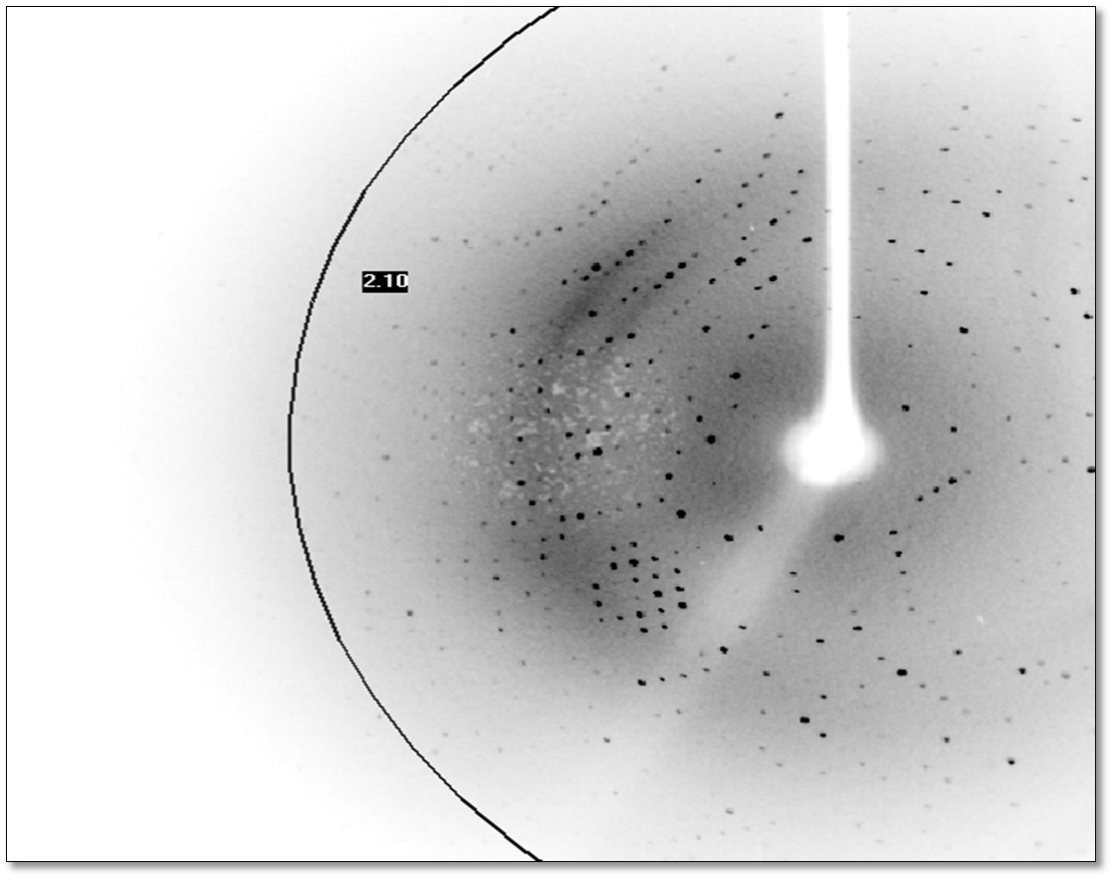
Diffraction patterns of thermostable mutant lipase D311E. The resolution is ~2.1 Å at the edge with starting position; distance: 50.00 mm, 2 theta: 22.00°, Omega: 31.64°, Phi: 271.48°, Chi: −30.01° and ending position; distance: 50.00 mm, 2 theta: 22.00°, Omega: 31.64°, Phi: 271.98°, Chi: −30.01°.

The D311E lipase crystal was diffracted at approximately 2.1 Å, and the diffraction pattern is shown in Figure 8. X-ray diffraction data for D311E lipase were collected using an in-house X-ray diffractometer. This crystal belonged to the *C2* space group with the unit-cell parameters *a* = 117.32 Å, *b* = 81.16 Å and *c* = 100.13 Å. Data processing statistics are shown in Table 4.

Generally, the volume of the crystal will affect the completeness (%) and the signal-to-noise ratio. The Matthews coefficient for the D311E lipase was 2.75 Å^3^Da^−1^ [28], and the crystals consisted of 55.33% solvent, which was in the range of 40–60% as stated in [29] with certain exceptions. A higher solvent content in a crystal significantly correlated with decreasing resolution.

Compared to BTL2 lipase, this crystal lipase was solved in closed conformation at high resolution (2.1 Å) using in-house X-ray diffractometer. In contrast, the diffraction data for BTL2 lipase crystal was collected using synchrotron radiation source at 2.2 Å [15].

### 2.7. Structural Analysis

The X-ray structure of D311E lipase (2.1 Å) showed a typical α/β hydrolase canonical fold consisting of 11 β-strands and 13 α-helices. The Ser113, Asp317 and His358 were assigned as the catalytic triad. The distance of charged side chains near the mutation site were measured to verify the possible ion pair formation. In D311E lipase, the ion pair network was composed of five amino acid residues (Arg 274, Thr 278, Gly 279, Arg 303 and Glu 311) connected by seven ion pairs. The comparison of the number of charged residues involved in ion pairs was made between D311E lipase with T1 lipase (Table 5). There were obvious differences in the ion pair distances at the mutation site. In D311E lipase, the distances from these ion pairs were between 2.5 Å and 5.2 Å. In contrast, the distances of ion pair involved in T1 lipase were longer than those in D311E lipase. On average, the distances indicated a strong interaction, which is around 2.0 Å to 3.0 Å. To date, there is no ion pair introduced at position D311 in lipase structure.

**Figure 9:**
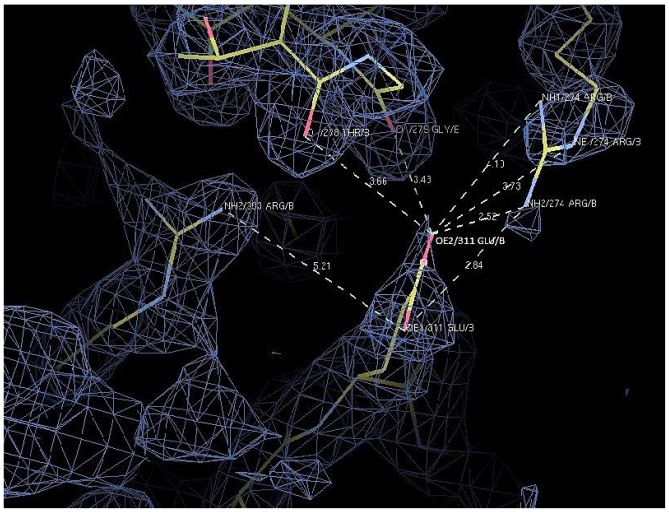
Snapshoot of ion pair interaction formation of D311E lipase crystal structure at residue between Glu311 and four residues (Arg 274, Thr 278, Gly 279 and Arg 303). * The generated 2Fo-Fc electron-density maps with 1.0 sigma level.

The Glu 311 and Gly 279 were connected by one hydrogen bond and linked to each other at an α-helix and a band region. This substitution affected the structural stability, which made the inter-connection of the D311E lipase (Figure 9). A significant increase in the number of ion pairs has been reported for most structures of thermostable proteins [30]. Furthermore, the ion pair interactions contributed to the forces that held the monomers together. For the glutamate dehydrogenase (GDH) from hyperthermophiles, the intersubunit ion pairs are involved in maintaining a stable structure [13]. In addition, the formation of ion-pair networks on the surface of the protein subunits that are buried at the interdomain and intersubunit interfaces may represent a major stabilizing feature that is associated with the adaptation of enzymes to extreme temperatures [31].

The coordinates of D311E crystal structure was deposited to RCSB Protein Data Bank under PDB ID code 3UMJ.

## 3. Experimental Section

### 3.1. Site Directed Mutagenesis

Two mutants were prepared: D311E, in which aspartic acid at position 311 for the inter-loop interaction study was changed to glutamic acid, and K344R, in which lysine at position 344 for the intra-loop interaction studies was mutated into arginine. The mutagenic plasmids constructed using the site-directed mutagenesis systems (Invitrogen) were introduced into the *Escherichia coli* BL21 (DE3) pLysS. Mutations were visualized and adjusted using Swiss PDB Viewer and the images were constructed using POV-Ray.

### 3.2. Prediction of Mutants Stability

The stability of putative mutants was predicted and performed using computational algorithm tools, namely I-Mutant 2.0. I-Mutant 2.0 is a support vector machine (SVM)-based tool for the automatic prediction of protein stability changes upon single amino acid substitutions [21]. The software computed the predicted free energy change value or sign (DDG), which is calculated from the unfolding Gibbs free energy value of the mutated protein minus the unfolding Gibbs free energy value of the native protein (kcal/mol). A positive DDG value indicates that the mutated protein possesses high stability whereas a negative DDG value indicates less stability of the mutant. A high reliability index (RI) is also important for interpreting the output data.

### 3.3. Protein Expression and Purification of T1 Lipase, D311E Lipase and K344R Lipase

**Figure 10:**
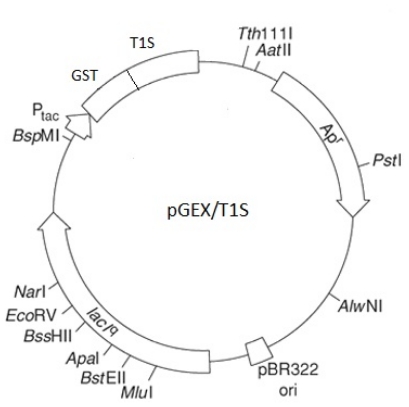
Schematic diagram of the parental plasmid pGEX/T1S. Expression of the T1, D311E and K344R lipase gene were done as the fusion protein.

The parental plasmid pGEX/T1S was used as the expression system (Figure 10). *E. coli* BL21 (DE3) pLysS mutagenic plasmid containing pGEX/T1, pGEX/D311E and pGEX/K344R were grown in Luria-Bertani (LB) medium that was supplemented with 100 μg/mL of ampicillin and 35 μg/mL of chloramphenicol under shaking conditions at 200 rpm and 37 °C. The expression of the lipases was induced using 25 μM of isopropyl β-d-1-thiogalactopyranoside (IPTG) at an OD_600_ value of approximately ~0.75. The cultures were grown for 12 h, and the pellets were harvested by centrifugation at 10,000 rpm for 10 min at 4 °C. The pellets were resuspended with 20 ml of phosphate-buffered saline (PBS, pH 7.4) that was supplemented with 5 mM of dithiothreitol (DTT) and sonicated (output: 2, duty cycle: 30) for 2 min. The crude enzymes were collected by centrifugation at 10, 000 *g* for 20 min at 4 °C. Filtered crude enzymes were loaded into a XK 16/20 column packed with 10 ml of Glutathione Sepharose 4 Fast Flow that had been pre-equilibrated with 10 column volumes (CV) of PBS (pH 7.4). The column was then washed with 3 CV of PBS (pH 7.4) and eluted with thrombin cleavage buffer (pH 8). The eluted fusion lipase fractions were pooled and incubated with thrombin enzymes to cleave the glutathione *S*-transferase (GST) tag. The GST tag was removed by applying the digested fusion lipase into second affinity chromatography columns packed with Glutathione Sepharose 4 Fast Flow, GSTrap and Hi-Trap Benzamidine, in which were attached in series [32]. An extra step of purification for D311E was performed to improve the quality of the crystal.

### 3.4. Circular Dichroism Spectral Analysis

All purified lipases in sodium phosphate buffer (10 mM, pH 8.0) were analyzed using the spectropolarimeter J-810 (Jasco, Japan) for circular dichroism (CD) spectral analysis. The warm-up periods of 50 °C to 80 °C and the wavelength scan of 180 nm to 250 nm were considered. The variable temperature measurement of T1, D311E and K344R lipases were performed using 10 mm cells after determining the CD value at 220 nm. The warm-up period was 50 °C to 80 °C, and the step was 1 degree per minute. The wavelength was set to 220 nm. The concentration was 1 mg/mL, and the top of the cell was completely closed using a cap. The data pitch, bandwidth, response, scanning speed, and accumulation were set to be 0.1 degree, 1 nm, 8 seconds, 1 degree per minute and 8 times, respectively.

### 3.5. Electrophoresis

SDS-PAGE and native PAGE were carried out on 12% running gel [27]. A broad range of protein standard (MBI Fermentas, St Leon-Rot, Germany) was used as a molecular mass marker.

### 3.6. Effect of Temperature on D311E and T1 Lipase Activity and Stability

The effect of temperature on D311E and T1 lipase activity was measured at temperatures ranging from 40 to 100 °C at 5 °C intervals for 30 min. The lipase activity was assayed at shaking rate of 200 rpm with olive oil as substrate [24].

Enzyme stability test was conducted by pre-incubating D311E and T1 lipase at 60 °C and 70 °C for various times prior to lipase assay at 70 °C under shaking condition (200 rpm) for 30 min.

### 3.7. Crystallization of D311E Lipase

A crystallization experiment was set up using the sitting drop vapor diffusion method. The D311E lipase was crystallized using the formulation 21 (0.1 M MES at pH 6.5, 0.1 M sodium phosphate, 0.1 M potassium phosphate and 2.0 M NaCl) of the Crystal Screen 2 reagent kit (Hampton Research, UK). The crystallization process was performed by mixing pure protein with the reservoir solution in a ratio of 1:1 (1.5 μL: 1.5 μL) using an Oryx8 protein crystallization robot (Douglas Instruments Ltd., UK) and then equilibrated with 50 μL of a reservoir solution at 20 °C for several days. After an overnight incubation, the crystal growth was observed using a stereomicroscope (Leica M165C, Germany).

### 3.8. X-Ray Data Collection

A set of X-ray data was obtained using the in-house Bruker X8 PROTEUM biological single crystal 103 diffractometer system with a MICROSTAR microfocus rotating anode generator 104 (Bruker. Germany). A PLATINUM 135 CCD detector was placed at a distance of 50 mm. Prior to performing the diffraction, the crystal was flash-cooled with cryoprotectant (40% glycerol and 1.9 M NaCl) to prevent ice crystal formation. The crystal was mounted under a liquid nitrogen flow at 100 K. The resolution data were indexed using PROTEUM and integrated with SAINT. SADABS was used to scale the data, and Xprep was used to determine the space group. The model was further built and refined using Refmac5 [33] and COOT, which is a molecular graphics application for manual model corrections [34].

## 4. Conclusions

In conclusion, the crystal structure of thermostable D311E lipase was solved at 2.1 Å. We showed that the introduction of an additional ion pair in the lipase structure increased the stability of the protein at high temperatures. The improved stability of D311E lipase was due to additional inter-loop interactions, which were indicated by the atomic details of D311E lipase with the observed additional ion pair and hydrogen bond interactions.

## Figures and Tables

**Table 1:** Summary of prediction energy, based on single point of mutation.

Mutant	Stability	Reliability Index (kcal/mole)	Relative Solvent Accessibility Area (%)
D311E	Increase	7.0	67.3
K344R	Decrease	4.0	39.9

**Table 2:** Thermodynamic parameters for the thermal denaturation transition of T1 lipase and its mutants as calculated from CD data.

Strain	*ΔS* (kJ/mol/K)	*ΔH* (kJ/mol)
T1	−1.22	−417.34
D311E	−1.88	−645.98
K344R	−1.83	−625.30

**Table 3:** Summary of the purification procedure for the thermostable D311E lipase.

Purification Steps	Total Activity (U)	Total Protein (mg/mL)	Specific Activity (U/mg)	Recovery (%)	Purification Fold
Crude	41,609.00	44.88	18.54	100.00	1.00
Affinity 1	18,366.50	21.67	19.78	44.14	1.83
Affinity 2	12,904.74	19.56	36.65	31.01	1.98
IEX	6,538.10	3.34	195.75	15.71	10.56

Note: The GST fusion lipase was purified under native condition. Affinity 1 represents Glutathione Sepharose HP, affinity 2 represents Glutathione-Sepharose HP, Glutathione-Sepharose 4FF and Benzamidine FF (high sub) attached in series, whereas IEX represents Q Sepharose FF.

**Table 4:** Summary of the crystallographic data.

	D311E
Unit cell parameters	*a* = 117.32 Å, *b* = 81.16 Å, *c* = 100.14 Å á = 90.00 °C, â = 96.49 °C, ã = 90.00 °C
Space group	*C2*
Wavelength (Å)	1.54
Resolution range (Å)	37.57–2.1 (2.2–2.1)
No. of observed reflections	249,261
No. of unique reflections	88,705
Redundancy (%)	2.93 (2.15)
Completeness (%)	96.9 (91.4)
Mean I/ó (I)	10.02 (4.01)
Molecules per asymmetric units	2
*V*_M_ (Å^3^ Da^−1^)	2.75
Solvent content (%)	55.33
*R*_merge_ (%)	8.33 (19.46)

**Refinement statistics**	

Structure solution method	Molecular replacement
Resolution (High)	2.1
Resolution (Low)	33.2
Cut-off sigma (F)	0.0
Number of reflections (Observed)	50139
Number of reflections (R-free)	2677
Percent reflections (Observed)	96.8
R-factor (Observed)	0.15
R-work	0.155
R-free	0.212

**Table 5:** The number of charged residues involved in ion pairs of T1 lipase and D311E lipase.

	Residue	Position	Residue	Position	Distance (Å)
T1	Asp 311	OD2	Arg 274	NH2	7.0
			Arg 274	NE	7.0
			Arg 274	NH1	8.8
			Thr 278	O	4.8
			Gly 279	O	4.9
D311E	Glu 311	OE1	Arg 303	NH2	5.2
			Arg 274	NH2	3.8
		OE2	Arg 274	NH2	2.5
			Arg 274	NE	3.7
			Arg 274	NH1	4.1
			Thr 278	O	3.7
			Gly 279	O	3.4
